# Association between health care access and food insecurity among lower-income older adults with multiple chronic conditions in Washington State, USA

**DOI:** 10.1017/S1368980022001240

**Published:** 2022-05-23

**Authors:** Courtney M Hill, Ashley S Tseng, Katherine Holzhauer, Alyson J Littman, Jessica C Jones-Smith

**Affiliations:** 1 Department of Epidemiology, University of Washington, Seattle, WA 98195, USA; 2 Seattle Epidemiologic Research and Information Center, Department of Veterans Affairs Puget Sound Health Care System, Seattle, WA, USA; 3 Seattle-Denver Center of Innovation for Veteran-Centered and Value-Driven Care, Health Services Research and Development, Department of Veterans Affairs Puget Sound Health Care System, Seattle, WA, USA; 4 Department of Health Systems and Population Health, University of Washington, Seattle, WA, USA

**Keywords:** Geriatrics, Food supply, Vulnerable populations, Healthcare disparities, Food insecurity, Chronic illness

## Abstract

**Objective:**

Lower-income older adults with multiple chronic conditions (MCC) are highly vulnerable to food insecurity. However, few studies have considered how health care access is related to food insecurity among older adults with MCC. The aims of this study were to examine associations between MCC and food insecurity, and, among older adults with MCC, between health care access and food insecurity.

**Design:**

Cross-sectional study data from the 2019 Behavioral Risk Factor Surveillance System survey.

**Setting:**

Washington State, USA.

**Participants:**

Lower-income adults, aged 50 years or older (*n* 2118). MCC was defined as having ≥ 2 of 11 possible conditions. Health care access comprised three variables (unable to afford seeing the doctor, no health care coverage and not having a primary care provider (PCP)). Food insecurity was defined as buying food that did not last and not having money to get more.

**Results:**

The overall prevalence of food insecurity was 26·0 % and was 1·50 times greater (95 % CI 1·16, 1·95) among participants with MCC compared to those without MCC. Among those with MCC (*n* 1580), inability to afford seeing a doctor was associated with food insecurity (prevalence ratio (PR) 1·83; 95 % CI 1·46, 2·28), but not having health insurance (PR 1·49; 95 % CI 0·98, 2·24) and not having a PCP (PR 1·10; 95 % CI 0·77, 1·57) were not.

**Conclusions:**

Inability to afford healthcare is related to food insecurity among older adults with MCC. Future work should focus on collecting longitudinal data that can clarify the temporal relationship between MCC and food insecurity.

The Food and Agricultural Organization of the United Nations defines food insecurity as lack of regular access to safe enough and nutritious food for normal growth and development and an active and healthy life^(1)^. In 2019, 10 % of households in the USA experienced food insecurity^(2)^, and one in five of these households included an older adult^(3)^. Food insecurity disproportionately affects low-income households^(2)^ and older adults with low income are particularly vulnerable to food insecurity due to factors such as social isolation, lack of transportation, disability and poor health^(4)^. Experiencing food insecurity is associated with malnourishment, poor chronic disease management^(5)^ and all-cause mortality^(6)^. Therefore, reducing food insecurity could improve overall health in this population.

Studies across North America have suggested that specific chronic conditions such as cancer, CVD, diabetes, high blood pressure and chronic lung disease are associated with food insecurity^(7–13)^. For older adults, burden of chronic conditions, measured by having multiple chronic conditions (MCC), has also been linked to higher levels of food insecurity^(14–16)^. Evaluating the extent to which MCC and food insecurity are associated is important considering that almost two-thirds of adults 65 years or older in the USA have MCC^(17)^. It has been hypothesised that food insecurity may contribute to chronic disease risk through stress and poor diet^(14,16)^, and that chronic disease may contribute to food insecurity among lower-income populations through high health care costs which leave less disposable income for food^(15)^. Additionally, having lower income or wealth is a common cause of both food insecurity^(18)^ and chronic disease^(19)^.

Unlike most other high-income nations, the USA lacks universal healthcare coverage^(20)^. Instead, the US health care system is unaffordable and inaccessible particularly for historically marginalised communities^(21)^. However, for individuals with MCC, the health care setting may be a critical venue for addressing unmet social determinants of health such as food insecurity^(22)^. In one US study, not having enough money for balanced meals was associated with lower health care access and quality^(23)^. For low-income individuals with MCC, the potential impact of health care access on food insecurity is twofold. First, health care coverage reduces personal health care spending which in turn may diminish the need to choose between health care and food. Second, access to health care may provide an opportunity to be screened for food insecurity^(24)^ and connected with resources that address food insecurity, such as the Supplemental Nutrition Assistance Program (SNAP) in the USA, meal programmes, fruit and vegetable financial incentive programmes or charitable food sources. Identifying which aspects of healthcare access is associated with food insecurity among older adults with MCC may inform more targeted screening methods and interventions to improve the health of a highly vulnerable population.

The first aim of this study was to investigate the relationship between MCC and food insecurity using data from the 2019 Washington State (WA) Behavioral Risk Factor Surveillance System (BRFSS) survey. We hypothesised that food insecurity would be more prevalent among lower-income older adults with MCC compared to lower-income older adults without MCC. The second aim of this study was to determine, among lower-income older adults with MCC, if health care access was associated with food insecurity. We hypothesised that food insecurity would be more prevalent among individuals with MCC and poorer health care access compared to those with MCC and better access. To our knowledge, no studies have examined the association between health care access and food insecurity among older adults with MCC.

## Methods

### Study design and data source

We used cross-sectional data from the 2019 WA BRFSS survey to examine the association between MCC and food insecurity among lower-income older adults (WA Department of Health, Center for Health Statistics, Behavioral Risk Factor Surveillance System, supported in part by the Centers for Disease Control and Prevention, Cooperative Agreement U58/DP006066–05 (2019)). BRFSS is an annual phone survey that samples non-institutionalised adults, 18 years or older, in the USA^(25)^. BRFSS includes core questions (standardised questions every state and territory are required to use each year), optional modules (standardised questions that states and territories can optionally use) and state-added questions (state-specific questions selected to reflect state health priorities). Data about chronic health conditions and healthcare use are collected as core questions each year and food insecurity data were collected as a new WA state-added question in 2019.

### Study population

Our study focused on lower-income older adults, 50 years or older, who lived in WA. Washington is a state in the northwest region of the USA and includes both highly populated metropolitan areas and rural agricultural areas. It is the thirteenth most-populated state in the USA with a population of more than 7·7 million people. Compared to the general USA, it has a higher socio-economic standing but the rural regions tend to have higher levels of poverty compared with the urban centers (about 20 % of the state population lives in rural regions) ^(26)^. We used annual household income as a percentage of the 2019 US Federal Poverty Level (% FPL)^(27)^ to define which participants were lower income. We set the upper bound of lower income at 250 % FPL^(28)^, corresponding to an annual income of $31 225 USD or less for an individual living alone in 2019. We excluded participants who had an income > 250 % FPL, missing responses to five or more of the chronic condition questions, or a missing response to the food insecurity question.

## Measures

### Exposures

There were two exposures of interest in this study. The first exposure was having MCC, which we defined as having ≥2 chronic conditions. BRFSS asks participants to self-report a history of eleven chronic health conditions: high blood pressure, high cholesterol, myocardial infarction, CHD, stroke, asthma, other cancers (excluding skin cancer), chronic obstructive pulmonary disease, kidney disease, diabetes and arthritis (Supplementary Table). For each condition, the participant was asked, ‘Have you EVER been told by a doctor, nurse or other health professional that you have/had (chronic condition)?’. Counting each affirmative response as one point, we created a variable for the total number of self-reported chronic conditions and then dichotomised it as has MCC (≥ 2) *v*. does not have MCC (0–1). The data source used for this study did not include items about medication use for every chronic condition we studied. However, a few of the chronic conditions, such as high blood pressure and high cholesterol, did ask about medication use. Within our study population that answered yes to either of these questions, 83·7 % and 62·4 % of the participants also responded yes to taking a medication for the condition which suggests that the majority of individuals with chronic conditions in the study were using medication.

The second exposure was healthcare access which was based on three questions: ‘Was there a time in the past 12 months when you needed to see a doctor but could not because of cost?’; ‘Do you have any kind of health care coverage, including health insurance, prepaid plans such as Health Maintenance Organisation, and government plans such as Medicare or Indian Health Service?’; and ‘Do you have one person you think of as your personal doctor or health care provider?’. Based on previous work^(29)^, the three healthcare access variables were coded so the exposed group would be poorer access: was *unable* to afford to see a doctor because of the cost *v*. was not unable to see a doctor because of cost; has *no* health care coverage *v*. has health care coverage; and does *not* have a primary care provider (PCP) *v*. has a PCP. In the USA, a PCP is a physician, nurse practitioner or physician assistant who has undergone primary care training. A PCP is the equivalent of a family practice or general practice physician in other countries.

### Outcome

The outcome was food insecurity and was assessed based on the question, ‘The food that I/we bought just didn’t last, and I/we didn’t have money to get more.’ Was that often, sometimes or never true for you in the last 12 months?’ Participants who responded ‘often true’ or ‘sometimes true’ were categorised as experiencing food insecurity and participants who responded ‘never true’ were categorised as not experiencing food insecurity. This question is the second item from a two-item screener previously validated against the eighteen-item USDA Food Security Survey Module in paediatric settings^(30)^. While it is customary to use both items from the screener to categorise individuals with an affirmative response to either item as food-insecure, our data source only used the second item. An affirmative response to the second item alone has a sensitivity of 82 % and a specificity of 95 %^(30)^.

### Confounders

We selected confounders *a priori* based on a minimum set of confounders identified using causal diagrams created separately for our two exposures (see online supplementary material, Supplemental Fig. 1 and 2). For our first exposure, MCC, we included the following confounders: sex, age, race/ethnicity, relationship status, educational attainment, employment status and annual household income as % FPL. For our analysis limited to those with MCC, we used the same set of confounders with the addition of metropolitan residence.

Sex was a binary variable (male, female) based on the BRFSS imputed version of the question, ‘Are you male or female?’. It is unclear whether survey participants answered their sex assigned at birth or gender identity^(31)^. We categorised age into two groups (50–64 years, ≥ 65 years). We categorised race/ethnicity as White Non-Hispanic (NH), Black NH, Asian NH, American Indian/Alaska Native NH, Hispanic, and Other NH. Relationship status was based on a question about marital status with six response options that we dichotomised into partnered (married, member of an unmarried couple) and not partnered (divorced, widowed, separated, and never married). Educational attainment was grouped into four categories (did not complete high school, high school degree or general equivalency diploma (GED), some college, college degree or higher). Employment status had three categories (employed, unemployed and retired), where ‘employed’ included those who were employed for wages or self-employed and ‘unemployed’ included those who were out of work, homemakers or those who were unable to work. We calculated annual household income as % FPL. The FPL is set annually by the US Department of Health and Human Services and is calculated based on household size^(27)^. Since BRFSS measures income as a categorical variable, we used a random uniform distribution to assign a continuous income value to each individual based on their income category. This method has been shown to accurately estimate % FPL in low-income populations^(32)^. Based on prior work^(8)^, we then categorised % FPL into five groups (< 51 %, 51–100 %, 101–130 %, 131–200 % and 201–250 %), with the additional delineation of the 201–250 % category to account for the eligibility cutoff for WA food assistance at < 200 % FPL^(33)^. Metropolitan residence (metropolitan and non-metropolitan) was based on the two-tiered rural-urban commuting area (RUCA) scale which was developed to compare population size and commuting patterns of different census tracts based on zip code^(34)^.

### Statistical analysis

We excluded any participants with ‘don’t know,’ ‘refused’ or missing responses for the confounder variables included in each analysis (available-case analysis). First, we conducted descriptive analyses by estimating the weighted prevalence of MCC in the total study population and within confounder subgroups. Weights were based on BRFSS survey design weighting that is performed to account for bias from selection probabilities, noncoverage and demographic differences between the sample population and the source population^(35)^. Then, we used modified Poisson regression^(36)^ to generate unadjusted prevalence ratios and confounder-adjusted prevalence ratios with 95 % CI. For our first aim, MCC was the exposure and food insecurity was the outcome. For our second aim, MCC was an inclusion criterion, poorer health care access was the exposure and food insecurity was the outcome. We chose modified Poisson regression because it is reliable with small sample sizes and accounts for overdispersion which is common in binary outcome data^(36)^. We used R version 4.0.3 for all statistical analyses.

## Results

There were 2326 lower-income older adults who participated in the WA BRFSS survey in 2019. All respondents answered five or more of the chronic condition questions. We excluded 208 (8·9 %) individuals because they had a missing response for food insecurity. Our final sample consisted of 2118 lower-income older adults.

Among the lower-income older adults living in WA in this study, 1580 had MCC (weighted prevalence = 81·8 %). Almost half (44·6 %) of study participants were male, 52·0 % were 50–64 years old and 44·7 % were partnered (Table 1). The majority of study participants were White NH (74·3 %), unemployed or retired (71·9 %), and resided in metropolitan areas (78·3 %). Respondents with MCC were more likely to be older, not partnered, unemployed or retired, and have an annual household income < 101 % FPL compared to those who did not have MCC.

**Table 1:** Characteristics of lower-income older adults* in Washington state, USA, by number of chronic conditions, behavioural risk factor surveillance system, 2019 (*n* 2118)

			Multiple chronic conditions†			
	Total (*n* 2118) Unweighted *n* (weighted %)		No (*n* 538) Unweighted *n* (weighted %)		Yes (*n* 1580) Unweighted *n* (weighted %)	
Characteristic	*n*	%	*n*	%	*n*	%
Sex‡						
Male	908	44·6	224	41·6	684	45·8
Female	1210	55·4	314	58·4	896	54·2
Age (years)						
50–64	858	52·0	254	58·7	604	49·5
≥ 65	1260	48·0	284	41·3	976	50·5
Race/Ethnicity						
White, Non-Hispanic	1788	74·3	456	74·8	1332	74·1
Black, Non-Hispanic	30	3·5	7	2·7	23	3·8
Asian, Non-Hispanic	32	5·7	9	6·4	23	5·5
American Indian/Alaska Native, Non-Hispanic	31	2·2	10	2·9	21	2·0
Hispanic	128	9·6	39	11·0	89	9
Other, Non-Hispanic	109	4·8	17	2·2	92	5·7
Relationship Status						
Partnered§	905	44·7	263	53·4	642	41·5
Not Partnered||	1213	55·3	275	46·6	938	58·5
Educational Attainment						
Did not complete high school	189	14·3	42	13·7	147	14·6
High school degree or GED	665	31·3	163	32·5	502	30·9
Some college	755	37·9	180	35·6	575	38·8
College degree or higher	509	16·4	153	18·1	356	15·7
Employment Status						
Employed	499	27·4	205	43·6	294	21·4
Unemployed¶	483	26·6	84	15·5	399	30·6
Retired	1129	45·3	246	39·0	883	47·7
Missing	7	0·7	3	2·0	4	0·2
Annual Household Income as % FPL**						
< 51 %	129	6·9	20	4·3	109	7·8
51–100 %	338	17·6	77	14·2	261	18·8
101–130 %	347	17·6	88	18·1	259	17·5
131–200 %	790	35·9	189	33·2	601	36·8
201–250 %	514	22·0	164	30·1	350	19·0
Metropolitan Residence ††						
Metropolitan	1338	78·3	335	76·5	1003	78·9
Non-Metropolitan	723	19·2	182	19·2	541	19·2
Missing	5·7	2·6	21	4·3	36	1·9

GED, general equivalency diploma; FPL, Federal Poverty Level.

* We defined lower-income as < 250 % FPL (corresponding to an annual income of $31 225 USD or less for an individual living alone in 2019) and included older adults who were at least 50 years old.

† At least two chronic conditions from a list eleven chronic conditions: high blood pressure, high cholesterol, myocardial infarction, CHD, stroke, asthma, any cancer (excluding skin cancer), chronic obstructive pulmonary disease, kidney disease, diabetes and arthritis.

‡ This binary variable is an imputed version of the question that asked ‘Are you male or female?’ on the Behavioral Risk Factor Surveillance System survey. It is unclear if a participant would answer the question based on their gender identity or sex assigned at birth.

§ Includes individuals who are married and members of an unmarried couple.

|| Includes individuals who are divorced, widowed, separated, and never married.

¶ Includes self-reported homemakers, students, or those unable to work.

** The FPL is set annually by the US Department of Health and Human Services and is calculated based on household size.

†† Derived from the Rural-Urban Commuting Area (RUCA).

The prevalence of food insecurity among all lower-income older adults in the study was 26·0 %. The prevalence of food insecurity was greater among lower-income older adults with MCC compared to those without MCC (29·1 % *v*. 17·5 %; prevalence ratio = 1·66; 95 % CI 1·26, 2·18) (Table 2). After adjusting for sex, age, race/ethnicity, relationship status, educational attainment, employment status and annual household income as % FPL, the prevalence of food insecurity was 1·50 times greater (95 % CI 1·16, 1·95) among participants with MCC compared to those without MCC.

**Table 2:** Weighted prevalence ratios for food insecurity by number of chronic conditions among lower-income older adults* in Washington state, USA, behavioral risk factor surveillance system, 2019

Multiple chronic conditions†	Food insecurity, weighted prevalence (%)	Unadjusted PR	95 % CI	Adjusted PR‡	95 % CI
No	17·5	Reference		Reference	
Yes	29·1	1·66	1·26, 2·18	1·50	1·16, 1·95

PR, prevalence ratio; % FPL, Percentage of the Federal Poverty Level.

The sample sizes for the unadjusted PR and the adjusted PR were 2118 and 2111, respectively.

* We defined lower-income as < 250 % FPL (corresponding to an annual income of $31 225 USD or less for an individual living alone in 2019) and included older adults who were at least 50 years old.

† At least two chronic conditions from a list eleven chronic conditions: high blood pressure, high cholesterol, myocardial infarction, CHD, stroke, asthma, any cancer (excluding skin cancer), chronic obstructive pulmonary disease, kidney disease, diabetes and arthritis.

‡ Adjusted for sex, age, race/ethnicity, relationship status, educational attainment, employment status and annual household income as %FPL.

Almost half of lower-income older adults with MCC who reported they were unable to afford visiting a doctor in the past 12 months also reported food insecurity compared to only 24·8 % of those who were able to afford a doctor in the past 12 months (adjusted prevalence ratio 1·83; 95 % CI 1·46, 2·28) (Table 3). The unadjusted prevalence ratio between not having health care coverage and food insecurity was 1·73 (95 % CI 1·26, 2·39). After adjusting for the confounders, the association was attenuated (adjusted prevalence ratio 1·49; 95 % CI 0·98, 2·24). The prevalence of food insecurity did not significantly differ between the lower-income older adults with MCC who did not and did have a PCP (adjusted prevalence ratio 1·10; 95 % CI 0·77, 1·57).

**Table 3:** Weighted prevalence ratios for food insecurity by healthcare access among lower-income older adults* with multiple chronic conditions† in Washington State, Behavioral Risk Factor Surveillance System, 2019

Healthcare access	Food insecurity, weighted prevalence (%)	Unadjusted PR	95 % CI	Adjusted PR‡	95 % CI
Unable to afford seeing a doctor in the past 12 months					
No	24·8	Reference		Reference	
Yes	49·9	2·02	1·61, 2·52	1·83	1·46, 2·28
Health care coverage					
Yes	27·8	Reference		Reference	
No	48·2	1·73	1·26, 2·39	1·49	0·98, 2·24
Has a primary health care provider					
Yes	28·3	Reference		Reference	
No	30·0	1·06	0·75, 1·50	1·10	0·77, 1·57

PR, prevalence ratio; %FPL, Percentage of the Federal Poverty Level.

For inability to afford seeing a doctor in the past 12 months, the sample sizes for the unadjusted PR and adjusted PR were 1575 and 1535, respectively. For health care coverage, the sample sizes for the unadjusted PR and adjusted PR were 1579 and 1539, respectively. For a primary health care provider, sample sizes for the unadjusted PR and adjusted PR were 1573 and 1533, respectively.

* We defined lower-income as < 250 % FPL (corresponding to an annual income of $31 225 USD or less for an individual living alone in 2019) and included older adults who were at least 50 years old.

† At least two chronic conditions from a list eleven chronic conditions: high blood pressure, high cholesterol, myocardial infarction, CHD, stroke, asthma, any cancer (excluding skin cancer), chronic obstructive pulmonary disease, kidney disease, diabetes and arthritis.

‡ Adjusted for sex, age, race/ethnicity, relationship status, educational attainment, employment status, annual household income as %FPL and metropolitan residence.

## Discussion

Among the lower-income older adults in this study, the prevalence of food insecurity was higher among participants who had MCC compared to those who did not have MCC. This finding is consistent with findings from other studies among low-income older adults^(14–16)^. The high healthcare and medication costs associated with having MCC may be one reason that this population faces a high burden of food insecurity. The association of MCC and food insecurity could also be bidirectional. Financial strains stemming from high healthcare costs could lead to food insecurity, and the poor nutrition resulting from food insecurity could also put individuals at a greater risk of developing chronic disease. Addressing the common systemic causes of food insecurity and chronic conditions (e.g. unaffordable health care, low-income or wealth, housing instability and structural racism) is crucial to alleviate the burden of food insecurity in lower-income older adults in the USA.

We found that being unable to afford to see a doctor was associated with food insecurity, which lends support to our hypothesis that financial insecurity could be a common cause of food insecurity and chronic conditions among older adults. Similar to our finding, at least two previous studies have found that food-insecure older adults were more likely to have cost-related non-adherence to medication compared to their food-secure counterparts^(37,38)^. The consequences of food-insecure individuals with MCC being unable to afford visiting a doctor and being non-adherent to medication potentially include exacerbation of existing chronic conditions and more severe impacts of chronic disease such as hospitalisation or death. Each of these consequences may contribute to increased personal health care spending and perpetuate a feedback loop of poor health, financial hardship and food insecurity. In addition to the financial costs of MCC, patients likely also face time and opportunity costs due to MCC, which may contribute to food insecurity. These include spending more time getting to doctor’s appointments and to the pharmacy to pick up prescriptions (i.e. transportation costs), inability to work full time or to find work that accommodates limitations imposed by medical conditions and chronic disease management tasks such as checking insulin for patients with diabetes^(39)^.

Contrary to our hypothesis, lacking health care coverage and having a PCP were not associated with food insecurity in adjusted analyses. To our knowledge, this is the first study to examine the association between health care insurance, having a PCP and food insecurity among older adults with MCC. There are a few possible explanations for why health care coverage was not statistically significantly related to food insecurity. First, individuals in our study who were 65 years or older (48·0 % of the study sample) have access to health care coverage through Medicare, the federal health insurance programme in the USA for all people who are 65 years or older which includes hospital and medical insurance. Medication coverage is an optional Medicare plan with additional costs. Since all individuals 65 years or older in this study were eligible for Medicare, our measure may not have been sensitive enough to detect whether an individual was able to get needed care when covered under Medicare. In addition, there is substantial variation in the quality and types of services provided by different health care plans within Medicare and across other or additional sources of coverage, which range from employer-based coverage, low-income coverage and coverage related to military service. Another potential explanation is that health care costs including medications are burdensome regardless of coverage status. This is consistent with previous work that suggested Medicare health coverage alone does not cover the cost of medications for older adults with chronic conditions^(37)^. Compared to a measure of the extent of health care coverage, any health care coverage may not be a good indicator of whether an individual has substantially reduced financial burden because of their health care coverage.

One potential reason why we did not find a significant association between not having a PCP and food insecurity could be that having a PCP does not impact the ability to afford food and does not operate on the monetary pathway between MCC and food insecurity we have hypothesised. Another potential reason is that participants’ PCP did not screen them for food insecurity. Previous work has shown that PCP do not routinely screen for food insecurity, and when they do, they prioritise screening in families with young children^(40,41)^. This is understandable given that screening for social determinants of health like food insecurity is a relatively new practice^(42)^. In addition, when PCP do screen for food insecurity, it is not clear whether they refer patients to services, how accessible those services are and whether patients would use the services. However, recent qualitative work has found that providers are receptive to incorporating screening measures for older adults and would be interested in referring patients to food assistance programs when necessary^(24)^. Future work should consider how older adult patients would respond to screening and referral to such programmes.

Almost 30 % of lower-income older adults with MCC in this study experienced food insecurity. Food insecurity among older adults is a growing problem as the population of older adults increases globally^(43)^. In addition, the COVID-19 pandemic led to an unprecedented increase in food insecurity among low-income families^(44)^. Potential interventions aimed at reducing existing food insecurity could include connecting lower-income older adults with MCC to food assistance programs and increasing funding for meal programs for older adults. Food assistance programs, such as SNAP in the USA, are effective at reducing food insecurity but are typically underutilised by older adults^(5)^ in part because of stigma around food assistance participation. However, further research about the barriers that older adults face in accessing support is warranted. Alternatively, universal meal programmes for older adults are an attractive option because they do not rely on meeting eligibility requirements like SNAP and may not have the same stigma^(45)^. These programmes have additional benefits such as home delivery of food which can overcome transportation and mobility challenges that older adults with chronic conditions face. In order for these programmes to be successful, adequate funding is necessary as current funding levels cannot provide for all older adults who are in need^(45)^.

While food assistance programmes and charitable food aid can be used to address existing food insecurity, they do not directly address the root causes of food insecurity nor do they prevent it. This would instead require system-wide changes^(46)^. In the USA, systems that propagate health disparities include unaffordable and inaccessible health care, low wages, housing instability and structural racism. Health care access interventions include providing coverage for people younger than 65 who cannot work due to MCC, lowering the age eligibility for Medicare to provide a smoother transition from prior employer-based or private health care coverage, reducing opportunity costs by improving clinic office hours and providing transportation to clinics and removing cost-related barriers to care such as choosing low-cost medications, limiting prescriptions for unnecessary medications or increasing insurance coverage for medications. Addressing other underlying causes of food insecurity will require dismantling inequitable systems and improving the social safety net for older adults. In addition, policies must take into account the life-long impacts of differential access to resources in order to support healthy aging.

### Limitations

There are several limitations to this study. First, the observational data are cross-sectional, which precludes our ability to determine the temporal relationships amongst variables of interest and potentiates that unmeasured confounders impacted the results. While our data provide evidence of the relationships, future studies should incorporate longitudinal data to investigate temporal relationships^(47)^ and collect information on additional potential confounders including food assistance use and social support provided by people other than partners. Second, food insecurity was a state-added question in WA in only a single year, so the sample size is relatively small and the study may not generalise to the entire USA or to individuals with MCC that could not be reached by phone. Although the prevalence of food insecurity among residents of WA is similar to that of the US population^(48)^, the health care system in WA may differ from other states and countries. Future work should consider these associations in locations with differing levels of health care access. Third, to measure food insecurity, our study used a single item from a two-item screener that was previously validated in a pediatric setting. The second item in the screener, which assesses worry about food insecurity, was not included. For this reason and because the screener is not validated in older adults, it is possible that food insecurity was underestimated in our study^(49)^. However, the prevalence of food insecurity in the study was similar to previous measurements which suggest the outcome may not have been meaningfully biased^(14)^. Finally, BRFSS is a self-reported survey conducted via telephone interviews, which may have resulted in misclassification for MCC due to recall bias or undiagnosed health conditions. This misclassification likely attenuated the observed association between having MCC and food insecurity.

These findings provide evidence that lower-income older adults with MCC have a high burden of food insecurity and that being unable to afford to see a doctor is related to food insecurity. Interventions could promote participation in food assistance programs through improved screening and referral programs for social determinants of health like food insecurity in the health care setting. In addition, food insecurity among older adults with MCC could be reduced by developing interventions aimed at reducing the financial and opportunity costs of health care. For instance, the age eligibility for Medicare participation could be lowered or Medicaid eligibility could be increased (i.e. Medicaid expansion). Introducing interventions to alleviate the burden of high health care costs in vulnerable populations could mitigate the high prevalence of food insecurity among lower-income older adults with MCC.

## Supplementary material

For supplementary material accompanying this paper visit https://doi.org/10.1017/S1368980022001240sup001. click here to view supplementary material

## References

[1] Food and Agriculture Organization of the United Nations (2021) Hunger and Food Insecurity. https://www.fao.org/hunger/en/ (accessed February 2022).

[2] Coleman-Jensen A , Rabbitt MP , Gregory CA et al. (2020) Household Food Security in the United States in 2019. United States Department of Agriculture. Economic Research Report no. 275. https://www.ers.usda.gov/webdocs/publications/99282/err-275.pdf?v=6959.9 (accessed May 2022).

[3] Hartline-Grafton H (2019) Hunger is a Health Issue for Older Adults: Food Security, Health, and the Federal Nutrition Programs. Food Research & Action Center. https://frac.org/wp-content/uploads/hunger-is-a-health-issue-for-older-adults-1.pdf (accessed February 2022).

[4] Vilar-Compte M , Gaitán-Rossi P & Pérez-Escamilla R (2017) Food insecurity measurement among older adults: implications for policy and food security governance. Glob Food Sec 14, 87–95.

[5] Gundersen C & Ziliak JP (2015) Food insecurity and health outcomes. Health Aff 34, 1830–1839. 10.1377/hlthaff.2015.0645 26526240

[6] Banerjee S , Radak T , Khubchandani J et al. (2021) Food insecurity and mortality in American adults: results From the NHANES-inked mortality study. Health Promot Pract 22, 204–214. 32748673 10.1177/1524839920945927

[7] Gucciardi E , Vogt JA , DeMelo M et al. (2009) Exploration of the relationship between household food insecurity and diabetes in Canada. Diabetes Care 32, 2218–2224. 19720843 10.2337/dc09-0823 PMC2782980

[8] Seligman HK , Laraia BA & Kushel MB (2010) Food insecurity is associated with chronic disease among low-income NHANES participants. J Nutr 140, 304–310. 20032485 10.3945/jn.109.112573 PMC2806885

[9] Laraia BA (2013) Food insecurity and chronic disease. Adv Nutr 4, 203–212. 23493536 10.3945/an.112.003277 PMC3649100

[10] Pérez-Escamilla R , Villalpando S , Shamah-Levy T et al. (2014) Household food insecurity, diabetes and hypertension among Mexican adults: results from Ensanut 2012. Salud Publica Mex 56, s62–s70. 25649455 10.21149/spm.v56s1.5167

[11] Gregory CA & Coleman-Jensen A (2017) Food Insecurity, Chronic Disease, and Health Among Working-Age Adults. United States Department of Agriculture. Economic Research Report no. 235. https://www.ers.usda.gov/webdocs/publications/84467/err-235.pdf?v=2483.2 (accessed May 2022).

[12] Charkhchi P , Fazeli Dehkordy S & Carlos RC (2018) Housing and food insecurity, care access, and health status among the chronically ill: an analysis of the Behavioral Risk Factor Surveillance System. J Gen Intern Med 33, 644–650. 29299816 10.1007/s11606-017-4255-z PMC5910337

[13] Mendy VL , Vargas R , Cannon-Smith G et al. (2018) Food insecurity and cardiovascular disease risk factors among Mississippi adults. Int J Environ Res Public Health 15, 2016. 30223555 10.3390/ijerph15092016 PMC6165024

[14] Jih J , Stijacic-Cenzer I , Seligman HK et al. (2018) Chronic disease burden predicts food insecurity among older adults. Public Health Nutr 21, 1737–1742. 29388533 10.1017/S1368980017004062 PMC6204426

[15] Leung CW , Kullgren JT , Malani PN et al. (2020) Food insecurity is associated with multiple chronic conditions and physical health status among older US adults. Prev Med Rep 20, 101211. 32983850 10.1016/j.pmedr.2020.101211 PMC7502278

[16] Tarasuk V , Mitchell A , McLaren L et al. (2013) Chronic physical and mental health conditions among adults may increase vulnerability to household food insecurity. J Nutr 143, 1785–1793. 23986364 10.3945/jn.113.178483

[17] Boersma P , Black LI & Ward BW (2020) Prevalence of multiple chronic conditions among US Adults, 2018. Prev Chronic Dis 17, 200130. 10.5888/pcd17.200130 PMC7553211 32945769

[18] Wight V , Kaushal N , Waldfogel J et al. (2014) Understanding the link between poverty and food insecurity among children: does the definition of poverty matter? J Child Poverty 20, 1–20. 25045244 10.1080/10796126.2014.891973 PMC4096937

[19] Oates GR , Jackson BE , Partridge EE et al. (2017) Sociodemographic patterns of chronic disease: how the mid-south region compares to the rest of the country. Am J Prev Med 52, S31–S39. 27989290 10.1016/j.amepre.2016.09.004 PMC5171223

[20] Weaver MR , Nandakumar V , Joffe J et al. (2021) Variation in health care access and quality among US states and high-income countries with universal health insurance coverage. JAMA Netw Open 4, e2114730. 34181011 10.1001/jamanetworkopen.2021.14730 PMC9434824

[21] Derose KP , Gresenz CR & Ringel JS (2011) Understanding disparities in health care access – and reducing them–through a focus on public health. Health Aff 30, 1844–1851. 10.1377/hlthaff.2011.0644 21976325

[22] Alley DE , Asomugha CN , Conway PH et al. (2016) Accountable health communities–addressing social needs through Medicare and Medicaid. N Engl J Med 374, 8–11. 26731305 10.1056/NEJMp1512532

[23] Cole MB & Nguyen KH (2020) Unmet social needs among low-income adults in the United States: associations with health care access and quality. Health Serv Res 55, 873–882. 32880945 10.1111/1475-6773.13555 PMC7518813

[24] Pooler JA , Hoffman VA & Karva FJ (2018) Primary care providers’ perspectives on screening older adult patients for food insecurity. J Aging Soc Policy 30, 1–23. 28768107 10.1080/08959420.2017.1363577

[25] Centers for Disease Control and Prevention (2021) About BRFSS. https://www.cdc.gov/brfss/about/index.htm (accessed February 2022).

[26] Washington State Department of Health (2014) Socioeconomic Position in Washington. https://www.doh.wa.gov/Portals/1/Documents/1500/Context-SEP-2014.pdf (accessed February 2022).

[27] United States Department of Health and Human Services (2022) 2019 Poverty Guidelines. https://aspe.hhs.gov/2019-poverty-guidelines (accessed February 2022).

[28] Silverman J , Krieger J , Kiefer M et al. (2015) The relationship between food insecurity and depression, diabetes distress and medication adherence among low-income patients with poorly-controlled diabetes. J Gen Intern Med 30, 1476–1480. 25917659 10.1007/s11606-015-3351-1 PMC4579205

[29] Fang J , Yang Q , Ayala C et al. (2014) Disparities in access to care among US adults with self-reported hypertension. Am J Hypertens 27, 1377–1386. 24847953 10.1093/ajh/hpu061 PMC4263941

[30] Hager ER , Quigg AM , Black MM et al. (2010) Development and validity of a 2-item screen to identify families at risk for food insecurity. Pediatrics 126, e26–e32. 20595453 10.1542/peds.2009-3146

[31] Moseson H , Zazanis N , Goldberg E et al. (2020) The imperative for transgender and gender nonbinary inclusion: beyond women’s health. Obstet Gynecol 135,1059–1068. 32282602 10.1097/AOG.0000000000003816 PMC7170432

[32] Hest R (2019) Four Methods for Calculating Income as a Percent of the Federal Poverty Guideline (FPG) in the Behavioral Risk Factor Surveillance System (BRFSS). University of Minnesota, State Health Access Data Assistance Center. https://www.shadac.org/sites/default/files/publications/Calculating_Income_as_PercentFPG_BRFSS.pdf (accessed May 2022).

[33] Washington State Department of Health (2021) SNAP-Ed in Washington. https://www.doh.wa.gov/CommunityandEnvironment/SNAPEd (accessed February 2022).

[34] United States Department of Agriculture, Economic Research Service (2020) Rural-Urban Commuting Area Codes. https://www.ers.usda.gov/data-products/rural-urban-commuting-area-codes.aspx (accessed February 2022).

[35] Centers for Disease Control and Prevention (2020) The Behavioral Risk Factor Surveillance System: Complex Sampling Weights and Preparing 2019 BRFSS Module Data for Analysis. https://www.cdc.gov/brfss/annual_data/2019/pdf/Complex-Smple-Weights-Prep-Module-Data-Analysis-2019-508.pdf (accessed February 2022).

[36] Zou G (2004) A modified poisson regression approach to prospective studies with binary data. Am J Epidemiol 159, 702–706. 15033648 10.1093/aje/kwh090

[37] Afulani P , Herman D , Coleman-Jensen A et al. (2015) Food insecurity and health outcomes among older adults: the role of cost-related medication underuse. J Nutr Gerontol Geriatr 34, 319–342. 26267444 10.1080/21551197.2015.1054575

[38] Bengle R , Sinnett S , Johnson T et al. (2010) Food insecurity is associated with cost-related medication non-adherence in community-dwelling, low-income older adults in Georgia. J Nutr Elder 29, 170–191. 20473811 10.1080/01639361003772400

[39] Syed ST , Gerber BS & Sharp LK (2013) Traveling towards disease: transportation barriers to health care access. J Community Health 38, 976–993. 23543372 10.1007/s10900-013-9681-1 PMC4265215

[40] Barnidge E , LaBarge G , Krupsky K et al. (2017) Screening for food insecurity in pediatric clinical settings: opportunities and barriers. J Community Health 42, 51–57. 27492774 10.1007/s10900-016-0229-z

[41] Hoisington AT , Braverman MT , Hargunani DE et al. (2012) Health care providers’ attention to food insecurity in households with children. Prev Med 55, 219–222. 22710141 10.1016/j.ypmed.2012.06.007

[42] Patil SP , Craven K & Kolasa K (2018) Food insecurity: how you can help your patients. Am Fam Physician 98,143–145. 30215895

[43] World Health Organization (2021) Ageing and Health. https://www.who.int/news-room/fact-sheets/detail/ageing-and-health (accessed February 2020).

[44] Wolfson JA & Leung CW (2020) Food insecurity during COVID-19: an acute crisis with long-term health implications. Am J Public Health 110, 1763–1765. 32970451 10.2105/AJPH.2020.305953 PMC7662000

[45] Gualtieri MC , Donley AM , Wright JD et al. (2018) Home delivered meals to older adults: a critical review of the literature. Home Healthc Now 36, 159–168. 29722706 10.1097/NHH.0000000000000665

[46] Tarasuk V (2011) A critical examination of community-based responses to household food insecurity in Canada. Health Educ Behav 28, 487. 10.1177/109019810102800408 11465158

[47] Noonan K , Corman H & Reichman NE (2016) Effects of maternal depression on family food insecurity. Econ Hum Biol 22, 201–215. 27281498 10.1016/j.ehb.2016.04.004

[48] United States Department of Agriculture, Economic Research Service (2016) Food Security and Nutrition Assistance. https://www.ers.usda.gov/data-products/ag-and-food-statistics-charting-the-essentials/food-security-and-nutrition-assistance/ (accessed February 2022).

[49] McKechnie R , Turrell G , Giskes K et al. (2018) Single-item measure of food insecurity used in the National Health Survey may underestimate prevalence in Australia. Aust N Z J Public Health 42, 389–395. 30035843 10.1111/1753-6405.12812

