# Supplementary material for: Association between health care access and food insecurity among lower-income older adults with multiple chronic conditions in Washington State, USA
Source: Public Health Nutr. 2022 May 23;26(1):199–207. doi: 10.1017/S1368980022001240 (PMC11077446; doi:10.1017/S1368980022001240)
Supplement: Supplementary file 1 [file S1368980022001240sup001.docx]

**SUPPLEMENTARY MATERIAL**

**Supplementary Table. Prevalence of chronic conditions of lower-income older adults* in Washington State, USA, by number of chronic conditions (N=2,118)**

| **Characteristic** | **Total**  **(N=2,118)**  **Unweighted n (weighted %)** | **Multiple Chronic Conditions**† | |
| --- | --- | --- | --- |
|  |  | **No**  **(N=538)**  **Unweighted n**  **(weighted %)** | **Yes**  **(N=1,580)**  **Unweighted n**  **(weighted %)** |

| **High Blood Pressure** |  |  |  |
| --- | --- | --- | --- |
| Yes | 1156 (52.1) | 80 (11.1) | 1076 (67.2) |
| No | 955 (47.5) | 458 (88.9) | 497 (32.2) |
| Missing | 7 (0.4) | 0 (0.0) | 7 (0.5) |
| **High Cholesterol** |  |  |  |
| Yes | 991 (47.6) | 67 (11.4) | 924 (61.0) |
| No | 1028 (48) | 471 (88.6) | 557 (33.0) |
| Missing | 99 (4.4) | 0 (0.0) | 99 (6.0) |
| **Myocardial Infarction** |  |  |  |
| Yes | 223 (10.1) | 3 (0.5) | 220 (13.6) |
| No | 1882 (88.9) | 535 (99.5) | 1347 (85.0) |
| Missing | 13 (1.1) | 0 (0.0) | 13 (1.4) |
| **Coronary Heart Disease** |  |  |  |
| Yes | 190 (8.3) | 1 (0.3) | 189 (11.2) |
| No | 1897 (90.2) | 537 (99.7) | 1360 (86.7) |
| Missing | 31 (1.5) | 0 (0.0) | 31 (2.0) |
| **Stroke** |  |  |  |
| Yes | 181 (8.2) | 4 (0.6) | 177 (11.0) |
| No | 1929 (91.4) | 534 (99.4) | 1395 (88.5) |
| Missing | 8 (0.4) | 0 (0.0) | 8 (0.5) |
| **Asthma** |  |  |  |
| Yes | 408 (19.9) | 21 (4.2) | 387 (25.7) |
| No | 1703 (79.9) | 517 (95.8) | 1186 (74.0) |
| Missing | 7 (0.2) | 0 (0.0) | 7 (0.2) |
| **Cancer, Excluding Skin** |  |  |  |
| Yes | 356 (16) | 25 (5.2) | 331 (20.0) |
| No | 1760 (83.8) | 513 (94.8) | 1247 (79.8) |
| Missing | 2 (0.1) | 0 (0.0) | 2 (0.2) |
| **Chronic Obstructive Pulmonary Disease** |  |  |  |
| Yes | 344 (15.9) | 8 (2.0) | 336 (21.0) |
| No | 1764 (83.7) | 530 (98.0) | 1234 (78.5) |
| Missing | 10 (0.4) | 0 (0.0) | 10 (0.5) |
| **Kidney Disease** |  |  |  |
| Yes | 150 (6.7) | 8 (1.6) | 142 (8.6) |
| No | 1959 (92.7) | 530 (98.4) | 1429 (90.6) |
| Missing | 9 (0.5) | 0 (0.0) | 9 (0.7) |
| **Diabetes** |  |  |  |
| Yes | 472 (23.1) | 14 (3.6) | 458 (30.3) |
| No | 1577 (73.1) | 524 (96.4) | 1053 (64.6) |
| Missing | 69 (3.8) | 0 (0.0) | 69 (5.1) |
| **Arthritis** |  |  |  |
| Yes | 1066 (49.1) | 92 (16.0) | 974 (61.3) |
| No | 1039 (50.4) | 446 (84.0) | 593 (38.1) |
| Missing | 13 (0.5) | 0 (0.0) | 13 (0.6) |

* We defined lower-income as <250% FPL (corresponding to an annual income of $31,225 USD or less for an individual living alone in 2019) and included older adults who were at least 50 years old.

† At least two chronic conditions from a list 11 chronic conditions: high blood pressure, high cholesterol, myocardial infarction, coronary heart disease, stroke, asthma, any cancer (excluding skin cancer), chronic obstructive pulmonary disease, kidney disease, diabetes, and arthritis.


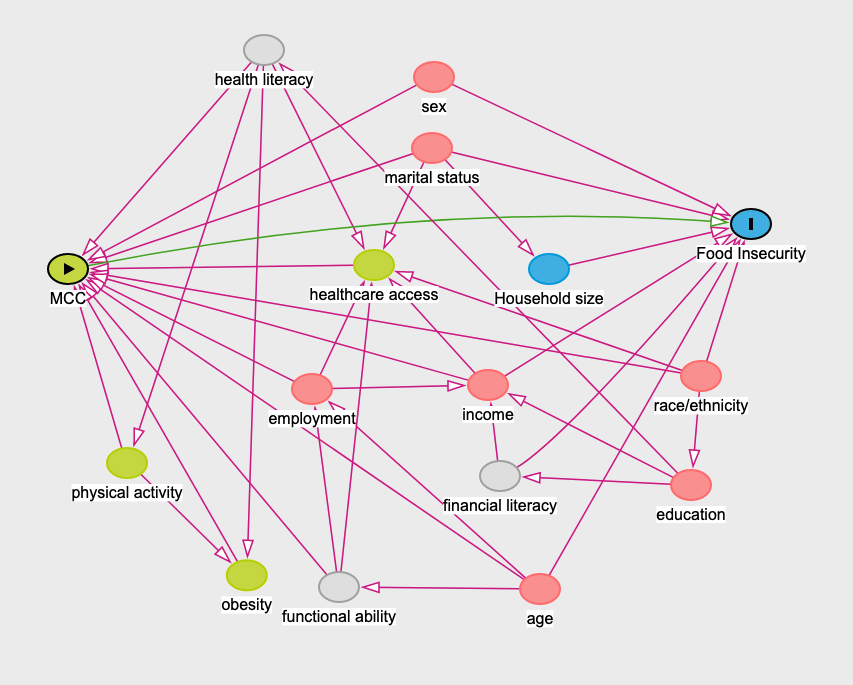


**Supplementary Figure 1. Causal diagram for the association between multiple chronic conditions (MCC) and food insecurity**

The green circle with the triangle indicates the exposure, the blue circle with the
vertical bar indicates the outcome. Green circles are variables that are ancestor of the exposure and blue circle are ancestors of the outcome. A pink circle represents variables that are ancestors of both the exposure and outcome (confounders). The grey circle represents an unobserved variable. The diagram was created using DAGitty (Johannes Textor, Benito van der Zander, Mark K. Gilthorpe, Maciej Liskiewicz, George T.H. Ellison. [Robust causal inference using directed acyclic graphs: the R package 'dagitty'.](http://dx.doi.org/10.1093/ije/dyw341) *International Journal of Epidemiology* 45(6):1887-1894, 2016.) Daggity suggests a sufficient minimum set of confounders.


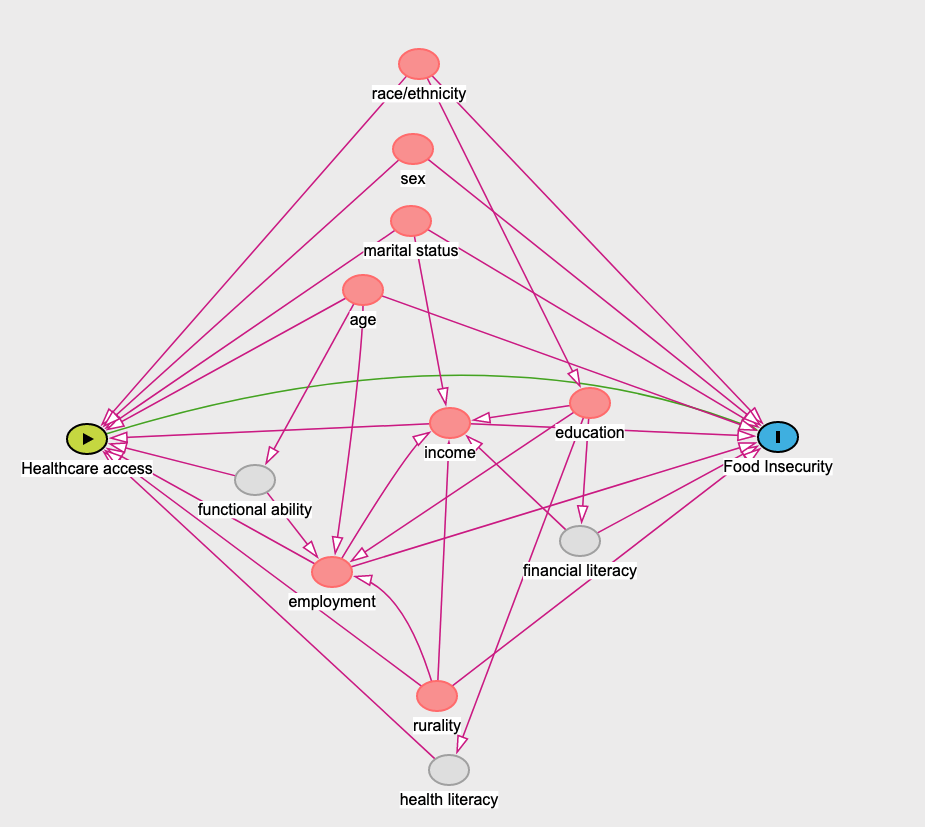


**Supplementary Figure 2. Causal diagram for the association between healthcare access and food insecurity**

The green circle with the triangle indicates the exposure, the blue circle with the
vertical bar indicates the outcome. Green circles are variables that are ancestor of the exposure and blue circle are ancestors of the outcome. A pink circle represents variables that are ancestors of both the exposure and outcome (confounders). The grey circle represents an unobserved variable. The diagram was created using DAGitty (Johannes Textor, Benito van der Zander, Mark K. Gilthorpe, Maciej Liskiewicz, George T.H. Ellison. [Robust causal inference using directed acyclic graphs: the R package 'dagitty'.](http://dx.doi.org/10.1093/ije/dyw341) *International Journal of Epidemiology* 45(6):1887-1894, 2016.) Daggity suggests a sufficient minimum set of confounders.
